# Beyond White-Nose Syndrome: Mitochondrial and Functional Genomics of *Pseudogymnoascus destructans*

**DOI:** 10.3390/jof11080550

**Published:** 2025-07-24

**Authors:** Ilia V. Popov, Svetoslav D. Todorov, Michael L. Chikindas, Koen Venema, Alexey M. Ermakov, Igor V. Popov

**Affiliations:** 1 Faculty “Bioengineering and Veterinary Medicine”, Don State Technical University, 344000 Rostov-on-Don, Russia; 2 ProBacLab, Laboratório de Microbiologia de Alimentos, Departamento de Alimentos e Nutrição Experimental, Food Research Center, Faculdade de Ciências Farmacêuticas, Universidade de São Paulo, São Paulo 05508-000, SP, Brazil; 3 CISAS-Center for Research and Development in Agrifood Systems and Sustainability, Instituto Politécnico de Viana do Castelo, 4900-347 Viana do Castelo, Portugal; 4 Health Promoting Naturals Laboratory, School of Environmental and Biological Sciences, Rutgers, The State University of New Jersey, New Brunswick, NJ 08901, USA; 5 Department of General Hygiene, I.M. Sechenov First Moscow State Medical University, 119435 Moscow, Russia; 6 Beneficial Microbes^®^ Consultancy, 6709 TN Wageningen, The Netherlands; 7 Division of Immunobiology and Biomedicine, Center of Genetics and Life Sciences, Sirius University of Science and Technology, 354340 Federal Territory Sirius, Russia

**Keywords:** white-nose syndrome, molecular evolution, evolutionary genomics, fungal evolution, phylogenetic comparative methods, phylogenomics

## Abstract

White-Nose Syndrome (WNS) has devastated insectivorous bat populations, particularly in North America, leading to severe ecological and economic consequences. Despite extensive research, many aspects of the evolutionary history, mitochondrial genome organization, and metabolic adaptations of its etiological agent, *Pseudogymnoascus destructans*, remain unexplored. Here, we present a multi-scale genomic analysis integrating pangenome reconstruction, phylogenetic inference, Bayesian divergence dating, comparative mitochondrial genomics, and refined functional annotation. Our divergence dating analysis reveals that *P. destructans* separated from its Antarctic relatives approximately 141 million years ago, before adapting to bat hibernacula in the Northern Hemisphere. Additionally, our refined functional annotation significantly expands the known functional landscape of *P. destructans*, revealing an extensive repertoire of previously uncharacterized proteins involved in carbohydrate metabolism and secondary metabolite biosynthesis—key processes that likely contribute to its pathogenic success. By providing new insights into the genomic basis of *P. destructans* adaptation and pathogenicity, our study refines the evolutionary framework of this fungal pathogen and creates the foundation for future research on WNS mitigation strategies.

## 1. Introduction

Bats play a crucial role in maintaining ecosystem stability, acting as pollinators, seed dispersers, and natural pest controllers [1,2]. However, in recent years, bat populations, particularly in North America, have faced an unprecedented decline due to WNS, a fungal disease caused by *Pseudogymnoascus destructans* [3,4]. Since its emergence in the United States in 2006, WNS has spread rapidly, leading to mortality rates exceeding 90% in some bat hibernacula [5]. The ecological consequences of this epidemic are profound: the loss of bat-mediated pest control has led to a 31% increase in insecticide use by farmers, correlating with an 8% rise in infant mortality rates in affected regions [6]. The epizootology of this disease is complex and depends on host susceptibility, environmental resistance, and pathogen virulence [7]. Despite extensive efforts to characterize *P. destructans*, fundamental gaps remain in our understanding of its genomic adaptations, pathogenicity mechanisms, and molecular evolution.

While *nuclear* genome analyses have provided insights into its gene content and potential virulence factors, *mitochondrial* genome evolution in this pathogen remains largely unexplored. Mitochondria play a central role in fungal adaptation, particularly in energy metabolism, oxidative stress response, and host–pathogen interactions [8,9]. In the emergence of fungal pathogens, mitochondrial genome rearrangements have been associated with shifts in virulence and environmental adaptation [10]. Yet the extent to which mitochondrial evolution contributes to the pathogenic success of *P. destructans* remains *terra incognita*.

Another major challenge in *P. destructans* research is the severe under-annotation of its genome. Despite the availability of high-quality genome assemblies [11], over 16,000 proteins in RefSeq remain classified as “uncharacterized”. This lack of functional annotation impedes efforts to identify critical metabolic pathways involved in host invasion, persistence, and proliferation. Recent advances in computational annotation methods, including orthology-based inference and metabolic pathway reconstruction, provide an opportunity to refine the functional landscape of *P. destructans*, shedding light on its pathogenic capabilities.

To address these gaps, we conducted a multi-scale genomic analysis of *P. destructans*, integrating pangenomics, phylogenetics, comparative mitochondrial genomics, and functional annotation. By reconstructing its evolutionary history within Leotiomycetes, we aimed to refine estimates of its divergence and explore potential links between mitochondrial genome evolution and pathogenic adaptation. Through comparative genomics, we sought to identify mitochondrial genome features unique to *P. destructans* and assess their implications for its biology. Finally, by employing an orthology-based functional annotation framework, we aimed to improve metabolic pathway resolution and uncover functional traits underlying its virulence and ecological success.

## 2. Materials and Methods

### 2.1. Pangenome Analysis of Mitochondrial Genomes in Leotiomycetes

To perform the pangenome analysis of mitochondrial genomes in Leotiomycetes, 24 mitochondrial genome sequences were retrieved from the RefSeq [12] database using Entrez-direct v. 22.4 [13] in FASTA format.

The analysis was conducted using the PanACoTA v.1.4.1 [14] toolkit. Pangenome construction was carried out using MMseqs2 v.17 [15] with a stringent protein identity threshold of 0.9 to focus on highly conserved genes, minimizing the impact of paralogs and ensuring robust phylogenetic signal, as recommended in high-resolution pangenome studies [16]. The pangenome structure was visualized using R packages ggplot2 (version 3.5.1) [17], dplyr, tidyr, and ggnewscale, generating: a donut chart illustrating the distribution of unique, shared, and core genes and a scatter plot depicting gene presence across analyzed genomes. Genes present in at least 66% of the genomes were extracted using MMseqs2 v.17, resulting in the selection of four genes for further analysis.

### 2.2. Phylogenetic Analysis

To reconstruct the evolutionary history of Leotiomycetes, we performed both gene-specific and phylogenomic analyses using mitochondrial coding sequences. As a preliminary control step, we inferred maximum likelihood (ML) phylogenies for four of the most conserved mitochondrial genes identified in the pangenome analysis. To extend this approach, we conducted a broader phylogenomic analysis based on 13 single-copy mitochondrial protein-coding genes (PCGs) shared across 19 Leotiomycetes species (*atp6*, *atp8*, *cox1*, *cox2*, *cox3*, *cob*, *nad1*, *nad2*, *nad3*, *nad4*, *nad4l*, *nad5,* and *nad6*), identified using Proteinortho v.6.3.4 [18].

For both approaches, multiple sequence alignments were generated using MAFFT v.7.525 [19], with poorly aligned regions removed using trimAl v.1.5.0 [20]. The best-fitting substitution models were selected via ModelFinder [21], and ML phylogenies were reconstructed using IQ-TREE v.2.4.0 [22] with 10,000 ultrafast bootstrap replicates [23]. The four gene-specific trees provided a reference framework for evaluating phylogenetic consistency, while the phylogenomic tree offered a higher-resolution view of species relationships within Leotiomycetes. All phylogenies were visualized using ggtree, incorporating species metadata retrieved from RefSeq via Phyloki v.0.5.51 (https://github.com/iliapopov17/phyloki accessed on 25 February 2025), ensuring a standardized and informative representation of taxonomic relationships.

### 2.3. Bayesian Evolutionary Analysis

To explore the evolutionary relationships and divergence times within Leotiomycetes, Bayesian phylogenetic analysis was conducted using BEAST v.1.10.4 [24]. The thirteen PCGs identified above were concatenated [25] and included in the analysis. *rps3* was not included in the analysis as no substitution rate estimates were available for this gene in the comparative mitochondrial study by Aguileta et al., and it is not part of the standard set used for evolutionary rate normalization [26].

The analysis was designed using BEAUti, where the mtREV substitution model was applied, along with a strict molecular clock. A lognormal prior was set on the clock rate, with a mean of 0.01 substitutions per site per million years and a standard deviation of 0.5 in real space, based on mitochondrial rearrangement-normalized substitution rates reported for Sordariomycetes [26]. While Leotiomycetes-specific rates are not available, their close phylogenetic relationship to Sordariomycetes—coupled with equivalent fossil-calibrated divergence times [27]—justifies using this rate as a proxy. A normal prior was applied to the root height, centered at 298 million years with a standard deviation of 10, based on fossil-constrained divergence estimates for the Leotiomycetes–Sordariomycetes split obtained from nuclear phylogenies using *18S*, *28S*, *RPB1*, and *RPB2* genes [27]. Despite originating from nuclear loci, these fossil-informed calibrations are appropriate for time-scaling mitochondrial phylogenies, as they constrain divergence time rather than substitution dynamics. The MCMC chain was run for 100,000,000 generations, sampling every 10,000 steps. Convergence and effective sample sizes (ESS) for all model parameters were assessed in Tracer v.1.7 [28], confirming sufficient sampling (ESS > 200). The maximum clade credibility (MCC) tree was summarized using TreeAnnotator with a 5% burn-in (burn-in as stated: 500) and node heights set to mean heights.

Final tree visualization and annotation were performed using ggtree and deeptime, incorporating species metadata retrieved in the previous step. To contextualize divergence events within a broader environmental framework, the tree was complemented with a 485-million-year reconstruction of Earth’s surface temperature, based on global mean surface temperature (GMST) estimates from Judd et al. (https://github.com/EJJudd/PhanDA/blob/main/5_Outputs/PhanDA_GMSTandCO2_percentiles.csv accessed on 25 February 2025) [29].

### 2.4. Comparative Genomic Analysis of Mitochondrial Genomes

Given the primary focus of this study on *P. destructans* and the close evolutionary relationship identified among four other fungal species, their mitochondrial genome sequences in FASTA format were used to calculate the Average Nucleotide Identity (ANI) using FastANI v.1.34 [30]. The resulting ANI values were visualized as a heatmap generated with pandas, seaborn, and matplotlib, providing a comparative assessment of genomic similarity among the selected *Leotiomycetes* representatives.

Additionally, their complete mitochondrial genomes were retrieved from RefSeq in GenBank (.gb) format using Entrez-direct. Genomic synteny analysis was conducted using pyGenomeViz v.1.5.0 (https://github.com/moshi4/pyGenomeViz accessed on 25 February 2025) and MMseqs2 v.17 to compare mitochondrial genome architecture across these species. To account for the circular nature of fungal mitochondrial DNA and to avoid artifactual rearrangement signals introduced by arbitrary starting positions, all mitochondrial genomes were linearized and anchored to the same conserved reference gene prior to synteny visualization. Functionally related genes were grouped accordingly during visualization to enhance interpretability.

To ensure a comprehensive comparative genomic analysis, we extended our approach by incorporating a biogeographic visualization of the studied mitochondrial genomes. This allowed us to examine the spatial distribution of the respective fungal species in relation to their evolutionary divergence. The map was constructed using temperature data from WorldClim [31] and geographic layers from Natural Earth (https://www.naturalearthdata.com accessed on 25 February 2025). Data processing and visualization were carried out in R v.4.4.2 (R Foundation for Statistical Computing, Vienna, Austria) with the sf, stars, terra, tmap, and dplyr packages. The Miller cylindrical projection was chosen for its suitability in representing high-latitude regions, including Antarctica [32]. Temperature data were processed and reprojected accordingly. Country boundaries and ocean features were sourced from the ne_110m_admin_0_countries and ne_110m_ocean layers of Natural Earth, ensuring accurate placement of geographic points. Data on species geographic distribution were retrieved from the Phyloki-generated metadata in the previous step.

### 2.5. Functional Annotation of Pseudogymnoascus destructans

To functionally annotate the proteome of *P. destructans*, protein sequences were retrieved from RefSeq using Entrez-direct. Uncharacterized proteins were obtained using the query “*P. destructans* AND Fungi AND uncharacterized AND srcdb_refseq[PROP]”, yielding 16,425 sequences. Characterized proteins were retrieved using the query “*P. destructans* AND Fungi NOT uncharacterized AND srcdb_refseq[PROP]”, resulting in 3909 sequences. All retrieved sequences were combined into a single dataset, generating three FASTA files: uncharacterized, characterized, and a complete coding sequence dataset.

Functional annotation was performed using eggNOG-mapper v.2.1.12 [33] within a Snakemake v.8.28.0 [34] pipeline to optimize computational efficiency. To ensure a systematic functional classification, redundant annotations were removed, retaining only unique entries. Each protein was assigned to a functional category based on its Clusters of Orthologous Genes (COG) classification, with multi-letter categories reduced to the first letter (e.g., COG4862 (KTN) was categorized under K). The functional distribution of *P. destructans* was analyzed by generating bar plots of absolute counts, separately for the complete dataset (uncharacterized + characterized proteins) and for the subset of characterized proteins. Additionally, a stacked bar chart was used to illustrate the relative representation of functional categories across three profiles: complete (all proteins), uncharacterized (only uncharacterized proteins), and characterized (only characterized proteins). Data visualization was performed in R using the tidyverse, patchwork, paletter, and forcats packages. To complement these results and assess annotation consistency, we additionally ran InterProScan v.5.66-97.0 [35], applying it to a complete coding sequence dataset.

## 3. Results

### 3.1. Pangenome Composition

**Figure 1:**
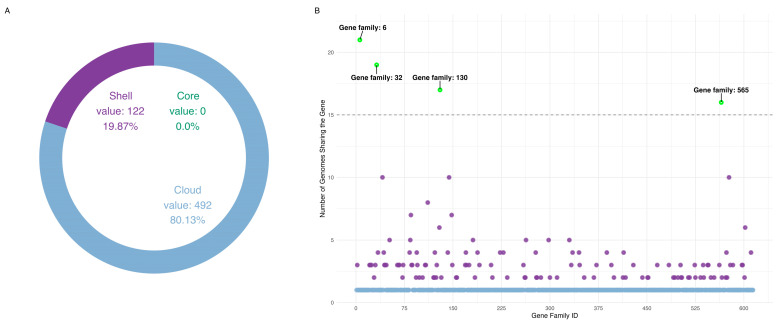
Composition of mitochondrial pangenome of Leotiomycetes: (**A**)—Distribution of sets of genes. The donut chart represents the proportion of cloud (blue), shell (purple), and core (dark green) genes. Given the absence of core genes, no corresponding segment is represented in the visualization. (**B**)—Scatter plot representation of gene family distribution in the pangenome. Each point represents a gene family, with the x-axis indicating gene families and the y-axis showing their presence across genomes. Cloud genes are marked in blue and shell genes are marked in purple. Genes exceeding the 15-genome threshold were retained for further analysis, with those marked in green.

The pangenome analysis of 24 Leotiomycetes mitochondrial genomes identified 614 gene families (Figure 1A), of which 492 (80.1%) were cloud, occurring in only a single genome. A total of 122 gene families (19.9%) were classified as shell, being present in more than one genome, but not universally conserved. Notably, no core genes—those present in all analyzed species—were detected. However, four gene families presented in more than 2/3 out of 24 genomes were detected (Figure 1B). These genes, while not universally conserved, exhibit broader distribution patterns compared to the rest of the pangenome.

Gene family ID 6 was annotated as *nad4l*, occurring in 21 genomes, making it the most conserved mitochondrial gene in our dataset. Gene family ID 32 was identified as *cox2*, gene family ID 130 corresponded to *cob*, and gene family ID 565 was assigned to *cox1*. These three genes were detected in 19, 17, and 16 mitochondrial genomes, respectively.

### 3.2. Phylogenetic Inference

**Figure 2:**
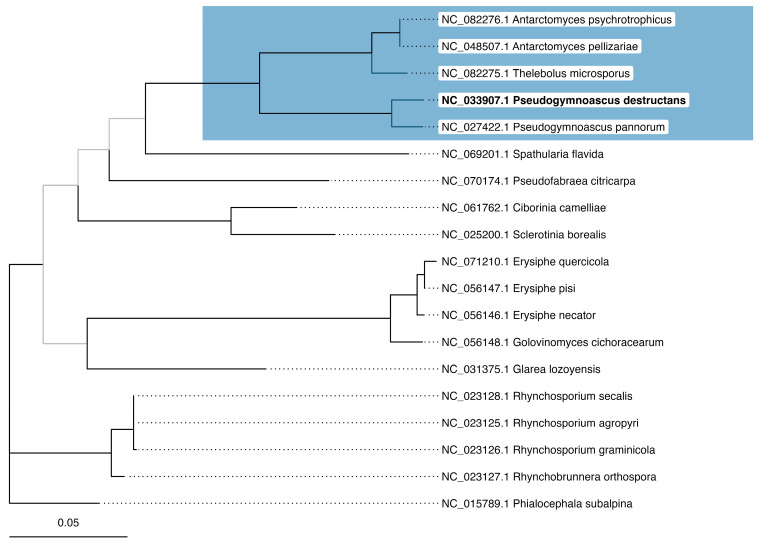
Maximum-likelihood phylogenetic tree based on the thirteen protein-coding genes. The tree was constructed based on *atp6*, *atp8*, *cox1*, *cox2*, *cox3*, *cob*, *nad1*, *nad2*, *nad3*, *nad4*, *nad4l*, *nad5,* and *nad6* genes under cpREV+F+R3 substitution model. The tree is midpoint rooted. Clades with bootstrap support <70 are shaded in gray. The clade containing *Pseudogymnoascus destructans* (bold leaf label) and its close relatives is highlighted in blue.

The phylogenomic tree constructed from 13 mitochondrial PCGs provides a robust framework for understanding evolutionary relationships within Leotiomycetes. A well-supported and distinct clade consistently included the five key species under investigation: *Pseudogymnoascus destructans* (NC_033907), *Pseudogymnoascus pannorum* (NC_027422), *Thelebolus microsporus* (NC_082275), *Antarctomyces pellizariae* (NC_048507), and *Antarctomyces psychrotrophicus* (NC_082276). This clustering reinforces their close evolutionary relationship within the class (Figure 2).

The majority of branches exhibited high bootstrap support, reflecting strong phylogenetic signal across the dataset. Only four nodes had bootstrap values under 70, suggesting reduced support in a few specific regions of the phylogeny. Nevertheless, the overall topology remained largely consistent with the reference maximum-likelihood trees based on individual, highly conserved mitochondrial genes (Figure S1). The consistency between the phylogenomic and gene-specific phylogenies further validates the inferred evolutionary relationships and supports the robustness of the phylogenetic framework. To complement this analysis, we also conducted a pangenome assessment across the same five-species clade using the pipeline described in the Materials and Methods, with the protein identity threshold set at 80% to reflect their close relatedness (Figure S2).

### 3.3. Bayesian Evolutionary Inference

**Figure 3:**
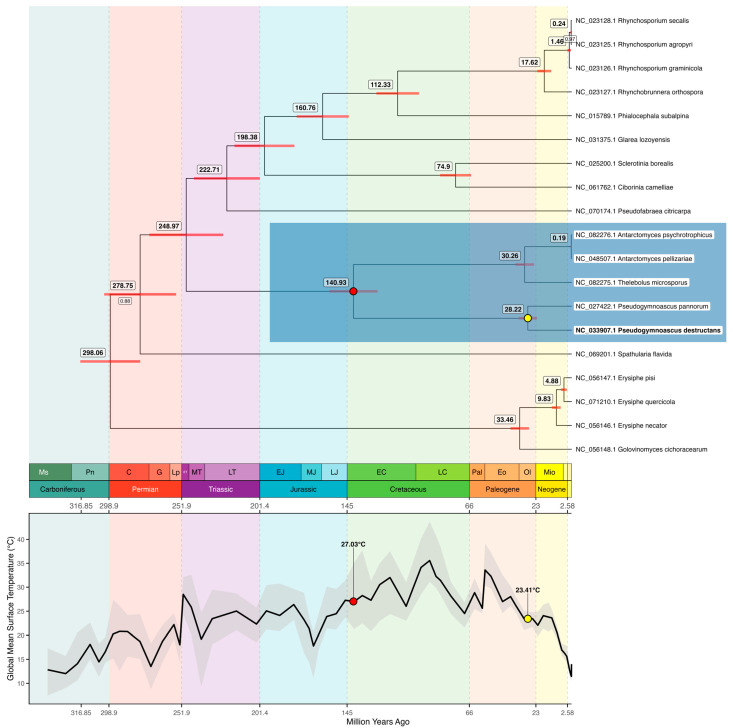
Bayesian phylogenetic tree of Leotiomycetes mitochondrial genomes with estimated divergence times. The clade containing *Pseudogymnoascus destructans* (bold leaf label) and its close relatives is highlighted in blue. Node ages (in million years) are shown to the upper left of each node, with 95% highest posterior density intervals represented by red horizontal bars. Posterior probabilities < 1.0 are displayed below corresponding branches. The top panel shows the phylogeny; the bottom panel displays the global mean surface temperature (GMST) curve. A red dot marks the estimated divergence time of the five species most recent common ancestor (MRCA), and a yellow dot indicates the MRCA of *P. destructans* and *Pseudogymnoascus pannorum*. Geological time scale with periods and epochs is shown for both tree and GMST curves. Ms = Mississippian, Pn = Pennsylvanian, C = Cisuralian, G = Guadalupian, Lp = Lopingian, ET = Early Triassic, MT = Middle Triassic, LT = Late Triassic, EJ = Early Jurassic, MJ = Middle Jurassic, LJ = Late Jurassic, EC = Early Cretaceous, LC = Late Cretaceous, Pal = Paleocene, Eo = Eocene, Ol = Oligocene, Mio = Miocene.

A Bayesian phylogenetic analysis was performed to estimate the evolutionary divergence times within Leotiomycetes, with a particular focus on the clade containing *P. destructans* and its closest relatives. The resulting time-calibrated phylogeny (Figure 3) largely recapitulates the topology observed in the ML trees, with *P. destructans* (NC_033907), *P. pannorum* (NC_027422), *T. microsporus* (NC_082275), *A. pellizariae* (NC_048507), and *A. psychrotrophicus* (NC_082276) forming a strongly supported monophyletic group. Remarkably, there are only two branches on the tree with a poor posterior probability parameter (<1).

Divergence time estimates indicate that the most recent common ancestor (MRCA) of *P. destructans* and *P. pannorum* existed approximately 28.2 million years ago (Mya; 95% highest posterior density (HPD): 22.32–34.17 Mya), during a period when GMST was approximately 23.4 °C. In contrast, the MRCA of *A. pellizariae* and *A. psychrotrophicus* is estimated at only 0.19 Mya, suggesting a relatively recent evolutionary split. The broader clade encompassing *T. microsporus*, *A. pellizariae*, and *A. psychrotrophicus* traces back to 30.3 Mya, while the MRCA of all five focal species dates to 140.9 Mya (95% HPD: 125.79–157.11 Mya), when GMST was approximately 27 °C. Between 140.9 and 28.2 Mya, GMST underwent several fluctuations—including steep rises and falls—but overall decreased by approximately 3.6 °C, highlighting a long-term global cooling trend across the evolutionary history of these lineages. The estimated divergence time for the entire Leotiomycetes lineage is 298 Mya (95% HPD: 278.11–316.85 Mya), reflecting deep evolutionary separation within the class.

### 3.4. Mitochondrial Genomic Comparison

**Figure 4:**
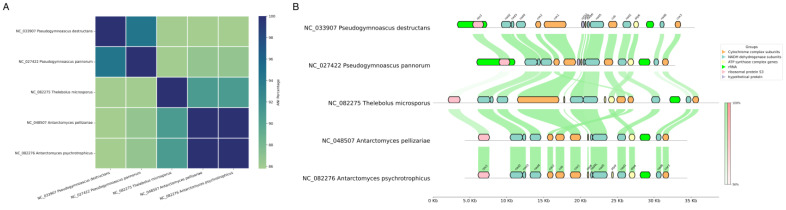
Comparative genomic visualization of mitochondrial genomes in five key Leotiomycetes species: (**A**)—Heatmap of pairwise Average Nucleotide Identity (ANI) among mitochondrial genomes. ANI values represent the percentage of nucleotide identity between each pair of mitochondrial genomes. Higher ANI values indicate greater sequence similarity. (**B**)—A linear comparison of mitochondrial genomes. Genes are color-coded according to their functional classification: Cytochrome complex subunits—orange, NADH dehydrogenase subunits—blue, ATP synthase complex genes—light yellow, rRNA—green, ribosomal protein S3—pink, and hypothetical proteins—light purple. A legend is provided for reference. Pairwise gene synteny is visualized using connecting lines that indicate sequence similarity, ranging from 56% to 100%. Green lines represent conserved gene orientation, while red lines indicate inversion events; however, no inversions were observed in the genomes analyzed.

To assess the degree of mitochondrial genomic similarity among the five focal species, a pairwise ANI analysis was conducted. The resulting heatmap (Figure 4A) provides a quantitative measure of nucleotide-level conservation across the mitochondrial genomes of *P. destructans*, *P. pannorum*, *T. microsporus*, *A. pellizariae*, and *A. psychrotrophicus*.

The highest ANI values were observed between *A. pellizariae* and *A. psychrotrophicus* (99.92%). In contrast, *P. destructans* and *P. pannorum* exhibit an ANI of 94.36%, indicating a closer relationship relative to the other species but still suggesting substantial genetic divergence. The mitochondrial genomes of *T. microsporus*, *A. pellizariae*, and *A. psychrotrophicus* show moderately high pairwise ANI values (~91%). Notably, *T. microsporus* exhibits a lower ANI (~85.9%) when compared to *P. destructans* and *P. pannorum*. These results provide further support for the phylogenetic structure observed in both ML and Bayesian analyses, where *Pseudogymnoascus* species form a distinct evolutionary unit, while *Thelebolus* and *Antarctomyces* species cluster separately with varying degrees of sequence conservation. The overall ANI trends emphasize the evolutionary divergence of these mitochondrial genomes, highlighting both recent speciation events and deeper phylogenetic separations within the group.

To evaluate mitochondrial genome organization and structural variation among the five focal species, gene synteny was visualized, highlighting large-scale genomic rearrangements and differences in overall genome size (Figure 4B). The mitochondrial genomes of *A. psychrotrophicus* and *A. pellizariae* exhibit near-identical gene content and synteny. Their mitochondrial genome sizes are almost equivalent (30,121 bp in *A. pellizariae* and 30,170 bp in *A. psychrotrophicus*), with only a minor discrepancy. The sole structural difference is the presence of an additional ATP synthase complex gene in *A. psychrotrophicus*, though its small size renders it functionally non-impactful at the genome-wide scale. This high degree of conservation suggests minimal evolutionary divergence at the mitochondrial level.

*T. microsporus* exhibits several structural variations relative to *Antarctomyces* species, primarily: an increase in genome size (38,803 bp); reduced sequence similarity in ribosomal protein S3 compared to *Antarctomyces*; a rearrangement of two genes within the Cytochrome complex subunits category; and a relocation of an ATP synthase complex gene within the genome.

*P. pannorum* possesses the smallest mitochondrial genome among the five species (26,918 bp), signifying extensive genome compaction. The following structural modifications distinguish it from *T. microsporus*: minor rearrangements within the Cytochrome complex subunits, ATP synthase complex genes, NADH dehydrogenase subunits, and a reduction in ribosomal protein S3 sequence similarity.

*P. destructans* has a mitochondrial genome of intermediate length (32,181 bp) and displays an overall gene order that is identical to that of *P. pannorum*. Their mitochondrial synteny is largely preserved, indicating a high degree of architectural conservation, which suggests that mitochondrial genome organization in *Pseudogymnoascus* species may be subject to structural constraint, with functional innovation occurring through mechanisms other than large-scale rearrangement.

The comparative analysis thus reveals a gradient of mitochondrial genome conservation: from the virtually identical architectures of the *Antarctomyces* species to the compact, yet syntenically stable genomes of the *Pseudogymnoascus* clade. Although genome sizes vary across lineages, pairwise sequence similarity across homologous genes remains consistently high, underscoring the overall conservation of mitochondrial coding regions within Leotiomycetes.

**Figure 5:**
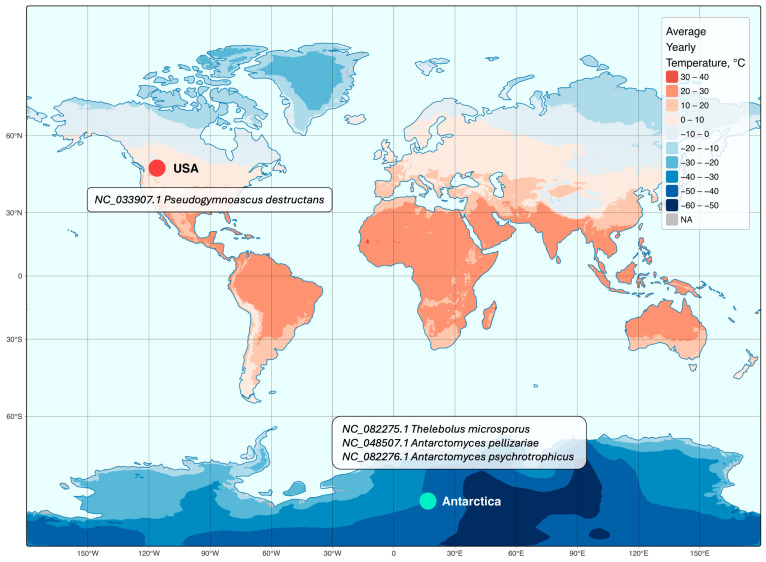
Geographic distribution of five analyzed fungal species. The map illustrates the locations of fungi in relation to global mean annual temperature. Light green dot represents Antarctica where *Thelebolus microsporus*, *Antarctomyces pellizariae,* and *Antarctomyces psychrotrophicus* are distributed. Red dot represents the United States of America, where *Pseudogymnoascus* fungi are prevalent. Country coordinates were extracted from the ne_110m_admin_0_countries dataset. The visualization was generated using the Miller cylindrical projection to account for high-latitude distortions. European and Asian locations of *Pseudogymnoascus destructans* are not presented due to the absence of mitochondrial genomic records of this fungus species from the RefSeq and GenBank databases. *Pseudogymnoascus pannorum* is not added to this map due to the lack of information on its isolation site in the corresponding RefSeq entry (NC_082275).

To complement the genomic comparisons, we examined the geographic distribution of the studied fungal species (Figure 5). The geographic assessment revealed a distinct biogeographic pattern: the understudied *P. destructans*’ mitochondrial genome originates from the United States, whereas *T. microsporus*, *A. pellizariae*, and *A. psychrotrophicus* are confined to Antarctica. The map visualization, constructed using the Miller cylindrical projection, integrates species occurrence data with global mean annual temperature patterns, providing a spatial context for the observed mitochondrial genome divergence.

### 3.5. Functional Profile of Pseudogymnoascus destructans

**Figure 6:**
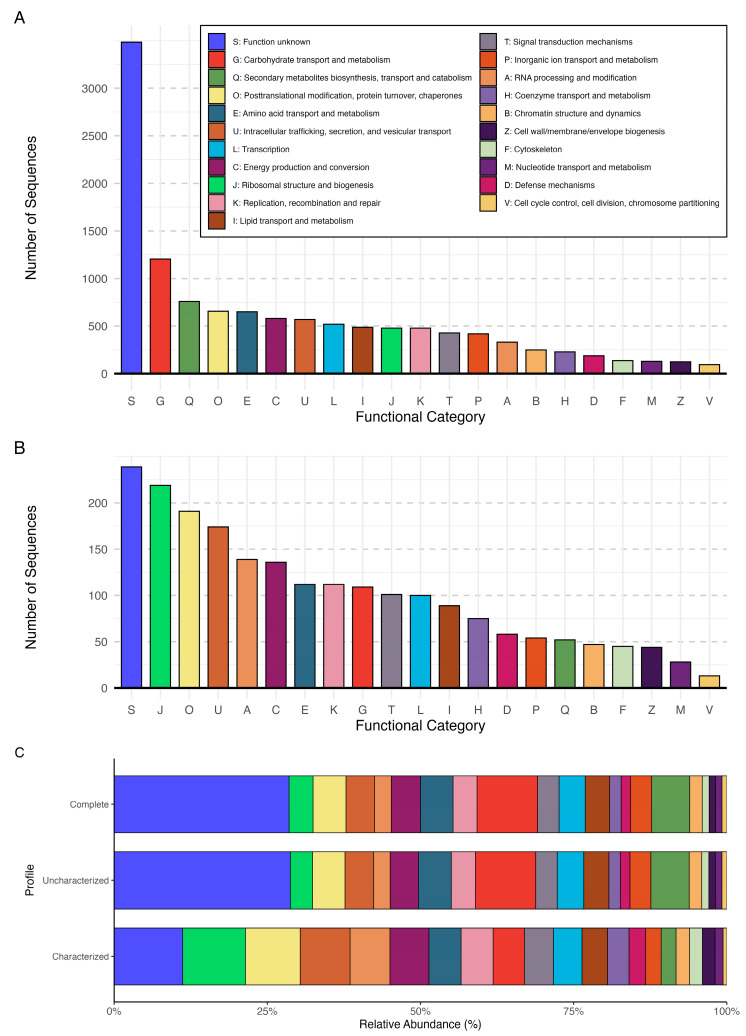
Functional profile of *Pseudogymnoascus destructans*. (**A**)—Barplot of complete *P. destructans* profile based on all the sequences from RefSeq. (**B**)—Barplot of *P. destructans* “characterized” profile based on “characterized” sequences from RefSeq. The x-axis lists COG categories; the y-axis denotes the number of functional elements in each category. (**C**)—Stacked barcharts of relative abundance of each COG category in 3 functional profiles: Complete (“uncharacterized” + “characterized” proteins), Uncharacterized (“uncharacterized” proteins only), and Characterized (“characterized” proteins only). The x-axis denotes the percentage of relative abundance; the y-axis lists functional profiles. All three plots share the same legend.

The functional annotation of *P. destructans* identified 12,206 proteins distributed across 21 COG functional categories, reflecting a diverse metabolic and cellular landscape (Figure 6A). While all categories contribute to the biological complexity of the fungus, we focused on the most abundant and biologically relevant groups, particularly those associated with metabolism, cellular processes, and information processing. The results presented here are based on eggNOG-mapper annotations, which are described in detail in the main text, while complementary InterProScan outputs are provided in the Supplementary Material.

#### 3.5.1. Information Storage and Processing

Several key categories involved in genome maintenance and regulation were identified. The transcriptional machinery was well represented (L: Transcription, 521 proteins), along with functional groups responsible for DNA replication, recombination, and repair (K, 478 proteins) and RNA processing (A, 332 proteins). Additionally, the presence of ribosomal biogenesis genes (J, 478 proteins) suggests an active protein synthesis system, crucial for rapid fungal growth and adaptation.

#### 3.5.2. Cellular Processes and Signaling

A significant portion of the *P. destructans* genome is dedicated to cellular homeostasis and stress adaptation. The posttranslational modification, protein turnover, and chaperone category (O, 656 proteins) is among the most enriched, likely reflecting the need for protein stability and folding under stressful conditions such as oxidative stress and host immune responses. Notably, the intracellular trafficking and secretion system (U, 570 proteins) is highly developed, potentially facilitating the export of virulence-associated proteins. Signal transduction (T, 428 proteins) plays a crucial role in environmental sensing, while defense mechanisms (D, 188 proteins) may contribute to fungal resilience against host defenses.

#### 3.5.3. Metabolism

The metabolic profile of *P. destructans* reveals a strong adaptation to its pathogenic lifestyle. Carbohydrate metabolism (G, 1206 proteins) is the most highly represented metabolic category, supporting efficient breakdown of host-derived carbon sources. Amino acid metabolism (E, 650 proteins) and lipid metabolism (I, 487 proteins) suggest metabolic flexibility, enabling survival in nutrient-limited environments such as bat hibernacula. Additionally, the presence of numerous secondary metabolite biosynthesis genes (Q, 760 proteins) may indicate the production of bioactive compounds, including potential virulence factors.

#### 3.5.4. Poorly Characterized Categories

A large fraction of proteins fell into categories with limited functional characterization. The “Function unknown” (S) category alone contained 3485 proteins, highlighting the significant gaps in annotation for *P. destructans*. Many of these uncharacterized proteins could play essential roles in fungal pathogenesis, warranting further investigation. While our analysis focused on the most highly represented and biologically relevant categories, additional functional classes, including those involved in cell cycle control (V, 96 proteins), chromatin remodeling (B, 249 proteins), and coenzyme metabolism (H, 229 proteins), further emphasize the complexity of this fungal pathogen.

#### 3.5.5. Comparison to Previously Characterized and Uncharacterized Proteins Dataset

The previously characterized protein subset (2137 proteins) encompassed all major functional categories observed in the complete profile but represented only 17.5% of the total functional landscape (12,206 vs. 2137 proteins) (Figure 6B). Characterized proteins were predominantly associated with essential cellular functions such as translation (J, 219 proteins), post-translational modification (O, 191 proteins), intracellular trafficking (U, 174 proteins), and energy production (C, 136 proteins). However, integrating previously uncharacterized proteins, identified using our refined analysis, revealed a markedly different functional landscape, exposing previously underestimated aspects of *P. destructans* metabolism. The complete dataset, based on our refined functional analysis, demonstrated a substantial expansion in carbohydrate metabolism (G, 1206 now vs. 109 proteins previously) and secondary metabolite biosynthesis (Q, 760 vs. 52 proteins), supporting the hypothesis that *P. destructans* employs diverse metabolic strategies to colonize host tissues. Amino acid metabolism (E, 650 vs. 112 proteins) and inorganic ion transport (P, 419 vs. 54 proteins) exhibited significant increases, suggesting an enhanced capacity to exploit nitrogen and metal ion resources in nutrient-limited environments. The enrichment of cellular defense mechanisms (D, 188 vs. 58 proteins) aligns with the pathogen’s need for resilience against host immune defenses. Furthermore, lipid metabolism (I, 487 vs. 89 proteins) and RNA processing (A, 332 vs. 139 proteins) showed substantial expansion, potentially contributing to membrane remodeling and regulatory adaptation under host-imposed stress conditions.

The relative proportion of “Function unknown” proteins (S) was lower in the characterized dataset compared to the complete profile (Figure 6C). However, this effect primarily results from the smaller total number of annotated genes rather than an actual enrichment of known functions. Notably, the uncharacterized protein subset (12,108 proteins) exhibited a functional distribution highly similar to the complete profile (Figure 6C), indicating that most metabolic insights were embedded within previously unannotated sequences.

## 4. Discussion

In this study, we conducted a multi-scale genomic analysis of *P. destructans* and its close relatives, employing an unconventional approach by performing a pangenome analysis at the class level. Pangenome analysis is most commonly conducted at the species or genus level [16], where lower genetic divergence facilitates clearer differentiation between core and accessory genes. As the taxonomic level rises, genetic divergence tends to increase, often leading to an almost complete loss of shared core genes [36], thereby reducing the effectiveness of conventional pangenome approaches. Additionally, while pangenome analyses have been widely applied to nuclear genomes, their use in mitochondrial studies remains limited due to inherent constraints—mitochondrial genomes are typically compact, encode a small number of essential genes, and exhibit strong functional conservation, all of which reduce the resolution and dynamic range typically captured in pangenomic comparisons [37]. Nevertheless, by applying a stringent protein identity threshold of 90%, we aimed to reduce spurious clustering across highly divergent taxa and ensure that only confidently conserved genes were retained. Using this approach, we successfully identified four mitochondrial genes shared by at least 66% of the analyzed genomes, providing a robust and phylogenetically meaningful core set. Among them, *nad4l* emerged as the most conserved, being present in 21 out of 24 species, even under the stringent similarity threshold. This finding is consistent with previous studies highlighting *nad4l* as one of the most conserved mitochondrial genes across Leotiomycetes fungi [38], reinforcing its phylogenetic utility. Thus, although our approach deviates from standard pangenome practices, it allowed us to systematically identify highly conserved genes within Leotiomycetes, providing a robust foundation for subsequent phylogenetic and evolutionary analyses. The phylogenetic trees reconstructed from these genes served as reference points, guiding the interpretation of broader phylogenomic and divergence dating analyses.

The phylogenomic analysis based on 13 mitochondrial genes revealed a well-supported evolutionary framework within Leotiomycetes. The ML tree demonstrated strong topological stability, with most branches receiving high bootstrap support. Notably, *P. destructans*, *P. pannorum*, *T. microsporus*, *A. pellizariae*, and *A. psychrotrophicus* consistently formed a distinct monophyletic clade, reinforcing their close evolutionary relationship. It is important to note that the results of this particular step of the study correspond to previous studies and are reproducible—they are fully consistent with the results of a study on comparative mitochondrial genomics of *Thelebolaceae* fungi obtained by Mi et al., 2024 [38], where an identical monophyletic group was observed. Despite the existence of a similar study, our results have a broader interpretation. While the previous study focused on the two newly sequenced mitochondrial genomes of *Thelebolaceae* fungi, the present study has a different aim: to analyze the genome of *P. destructans* in as much detail as possible. By evaluating the topology of the obtained trees, we were able to decide on the design of the next step of the study. In addition, all further steps were carried out for the first time.

The comparative analysis of mitochondrial genomes within P. destructans and its closest relatives reveals striking sequence conservation coupled with unexpected structural stability. The ANI values among the five fungal species remain strikingly high, with a minimum of 85.7%, reinforcing the close phylogenetic relationship previously established. The highest ANI values (99.92%) observed between *A. pellizariae* and *A. psychrotrophicus* reflect their recent divergence, while the moderate ANI between *Pseudogymnoascus* and *Thelebolus* species (~85.9 to 90.9%) supports a more ancient common ancestry.

A key question arising from our findings is why *P. destructans* shows no significant mitochondrial genome rearrangements despite its emergence as a bat pathogen. Mitochondrial genomes play a critical role in fungal pathogenicity by influencing energy production, stress tolerance, and virulence factor expression [39]. Widespread mitochondrial rearrangements may represent an adaptive shift linked to the transition from a free-living or psychrophilic lifestyle to host-dependent pathogenicity [40]. These rearrangements are often associated with high rates of homologous recombination [41], mobile element activity [42,43], or shifts in selective pressures that favor genomic plasticity [9,44,45]. In pathogenic fungi, drastic mitochondrial genome restructuring has been linked to adaptive responses, including metabolic flexibility [46], oxidative stress tolerance, and host–pathogen interactions [8,47]. For example, Ward et al. documented that *Ophiocordyceps* fungi undergoing a transition from free-living pathogens to insect symbionts exhibited alterations in both mitochondrial and nuclear genomes – such as gene loss and intensified selective pressures – corresponding to changes in their parasitic strategy [48]. Likewise, some human pathogens, such as *Cryptococcus* [49] and *Candida* [50], exhibit mitochondrial genome rearrangements associated with host adaptation and metabolic shifts. In contrast, *P. destructans* maintains a mitochondrial gene synteny highly similar to its psychrophilic relatives. Four of the analyzed species are non-pathogenic and primarily found in extreme cold environments, such as Antarctica (*A. pellizariae*, *A. psychrotrophicus*, *T. microsporus*) [51,52] or Canada (*P. pannorum*) [53]. These psychrophilic species share near-identical mitochondrial architectures, and *P. destructans*, despite its pathogenic lifestyle and temperate cave habitat across North America, Europe, and Asia, appears to retain this conserved ancestral gene order. This stability is notable given that fungal mitogenomes are often prone to rearrangement via recombination [40]. The absence of widespread mtDNA reorganization in *P. destructans* suggests that its adaptation to bats relied on different evolutionary routes. One possibility is that *P. destructans* evolved through metabolic streamlining rather than organelle genome reshuffling. Indeed, compared to its soil-dwelling relative *P. pannorum*, *P. destructans* has shed numerous metabolic functions and can utilize far fewer substrates [53]. This reduced, specialist metabolism aligns with a host-restricted niche and may have obviated any need for mitochondrial gene order changes. Unlike most fungal pathogens, *P. destructans* thrives at 4–15 °C during its host’s hibernation. It primarily infects superficial skin tissues in a two-phase (biotrophic-to-necrotrophic) manner reminiscent of plant pathogens [54], a strategy that could be executed without novel mitochondrial reconfiguration. Notably, *P. destructans* belongs to the Leotiomycetes, a lineage dominated by plant-associated fungi [55], and likely inherited effective virulence mechanisms (e.g. nutrienttropism and appressorium-like penetration structures) from that ancestry. This evolutionary head start may have reduced pressure to alter its mitochondrial genome when it jumped to animal hosts. Consistent with this idea, *P. destructans* is phylogenetically distant from other animal pathogens [54] and may reproduce clonally [56], limiting opportunities for the recombination events [57] that often drive mitochondrial rearrangements [58]. The lack of mitochondrial rearrangement in *P. destructans* highlights that pathogenic success can emerge via genomic streamlining and repurposing of existing traits rather than through organelle genome plasticity. In sum, *P. destructans* exemplifies a pathogen that has evolved a lethal host-specific lifestyle while paradoxically retaining a static mitochondrial genome. This suggests that strong selection can favor maintaining mitochondrial integrity when other adaptive strategies (gene loss, metabolic specialization, and host mimicry of plant-pathogen tactics) sufficiently meet the demands of a new ecological niche.

Our mitochondrial Bayesian evolutionary analysis provides a refined evolutionary framework for *P. destructans* and its closest relatives. A strongly supported clade consisting of *P. destructans*, *P. pannorum*, *T. microsporus*, *A. pellizariae*, and *A. psychrotrophicus* shares a common ancestor dating to approximately 141 million years ago, a time corresponding to a globally warm climate (GMST ≈ 27 °C). Between this ancestral divergence and the more recent split of *P. destructans* from *P. pannorum* (~28.2 Mya; 95% HPD: 22.32–34.17 Mya), global mean surface temperatures dropped by roughly 3.6 °C, although this interval was marked by significant climatic variability. Notably, this lineage endured and diversified through multiple climatic shifts, ultimately spreading across Antarctica and cold regions of the Northern Hemisphere. This is highly consistent with the 23.5 Mya estimate reported by Palmer et al., based on nuclear genome data, despite variations in analytical methods and taxon selection [58]. Notably, key Leotiomycetes such as *Antarctomyces* and *Thelebolus* were absent from Palmer’s dataset, while our analysis includes these taxa—offering a broader and complementary perspective. Together, these studies provide coherent insights into the evolutionary timing of this clade. The deeper divergence of *P. destructans* from its Antarctic relatives suggests long-term adaptation across hemispheres and environments, with potential ecological flexibility rooted in a cold-adapted ancestral lineage. Despite relying on a proxy substitution rate derived from Sordariomycetes, the estimate of a 141 Mya divergence remains credible, as our results are in excellent agreement with Palmer’s, who used nuclear genome data and reached similar conclusions at an important time point. We estimate the origin of Leotiomycetes at 298 Mya (95% HPD: 278.11–316.85 Mya). This is consistent with fossil-calibrated estimates by Beimforde et al., who placed the Leotiomycetes–Sordariomycetes split at 287 Mya (Calibration 1) and 309 Mya (Calibration 2) [27]. Since our prior root height was directly informed by these fossil-based nuclear calibrations, the observed concordance between our mitochondrial estimates and published nuclear timelines further strengthens the internal consistency and reliability of our model.

Functional annotation of *P. destructans* has been significantly expanded in this study, revealing a much broader functional landscape than previously recognized. While in the previously characterized proteins dataset, our annotation allowed us to assign 2137 COG categories, our reannotation incorporating uncharacterized proteins increased this number nearly sixfold to 12,206 proteins, providing a more comprehensive overview of the pathogen’s functional potential. This result suggests that a substantial portion of *P. destructans*’ functional diversity remained obscured due to the incomplete annotation of its genome. However, despite this progress, 3485 proteins remain classified as “Function unknown”, reflecting the broader challenge of deciphering fungal protein functions at a large scale. The under-annotation of fungal genomes is not solely a consequence of limited experimental validation but also of methodological constraints [59]. As a result, key metabolic traits in fungi, including those relevant to pathogenicity, may be underrepresented or misclassified. Expanding curated fungal-specific annotation resources is crucial for improving the accuracy of functional predictions and advancing our understanding of fungal pathobiology.

Despite the severity of WNS in the United States and Canada, the genome of its etiological agent, *P. destructans*, remains poorly characterized. As of now, the RefSeq database contains 16,425 protein sequences from *P. destructans* labeled as “uncharacterized protein”, highlighting the significant gaps in the functional annotation. Bioinformatics-driven studies on the fungal genome are notably scarce. One such study by Davy et al. demonstrated that *P. destructans* maintains stable gene expression when growing on the wing tissue of different bat species, showing no transcriptional shifts between superficial colonization and invasive tissue penetration [60]. Additionally, Forsythe & Xu sequenced and annotated the complete mitochondrial genome of *P. destructans*, which served as a key reference in our study [61]. We anticipate that our findings will provide researchers with valuable insights into the genomic and metabolic landscape of *P. destructans*, ultimately aiding in the development of strategies to mitigate WNS [62]. Despite the comprehensive genomic analysis conducted in this study, we must acknowledge the limitation of this analysis, which is the lack of mitochondrial genomic records of *P. destructans* isolates from Eurasian bat populations due to the absence of these records in publicly available datasets. This limitation naturally constrains the biogeographic depth of our analysis and limits the exploration of regional variation in mitochondrial genome structure. Nonetheless, it emphasizes the need for future studies to incorporate geographically diverse isolates and to complement genomic analyses with functional validation. Further work should include transcriptomic profiling under ecologically relevant conditions, cross-continental comparative genomics, and targeted functional assays to investigate the roles of candidate virulence genes.

## Data Availability

All genomic data of Leotiomycetes and *P. destructans* analyzed are available at NCBI and are provided together via Zenodo at https://zenodo.org/records/15045007 (accessed on 20 July 2025). Data generated in this study including pangenome composition, phylogenies, and manual annotations (that is, sequence files, alignments and tree files, compositions of orthogroups and sister groups, and so on) are also available via Zenodo. Public databases are available as follows: eggNOG annotations were obtained with eggNOG-mapper 2.1.12 (https://github.com/eggnogdb/eggnog-mapper accessed on 25 February 2025), and the NCBI genomes were downloaded in February 2025 (https://www.ncbi.nlm.nih.gov/refseq/ accessed on 25 February 2025). The pipeline used for the bioinformatic data analysis has been deposited in GitHub: https://github.com/PopovIILab/beyondWNS (accessed on 20 July 2025). This pipeline includes all the lab journals, scripts, and environments for each step of the study.

## Supplementary Materials

The following supporting information can be downloaded at: https://www.mdpi.com/article/10.3390/jof11080550/s1. Figure S1: Maximum-likelihood phylogenetic trees inferred on 4 highly conserved genes. A—The *nad4l* tree (mtZOA substitution model). B—The *cox2* tree (Q.plant+G4 substitution model). C—The *cob* tree (mtZOA+G4 substitution model). D—The *cox1* tree (mtZOA+R2 substitution model). All trees are midpoint rooted. Clades with bootstrap support < 70 are shaded in gray. Clades containing *Pseudogymnoascus destructans* (bold leaf label) and its close relatives are highlighted in blue; Figure S2: Composition of mitochondrial pangenome of *Pseudogymnoascus* and *Thelebolaceae* fungi. A—Distribution of sets of genes. The donut chart represents the proportion of cloud (blue), shell (purple), and core (dark green) genes. B—Scatter plot representation of gene family distribution in the pangenome. Each point represents a gene family, with the x-axis indicating gene families and the y-axis showing their presence across genomes. Cloud genes are marked in blue and shell genes are marked in purple. Core genes marked in green. Gene family 1 corresponds to *nad3*, 2—*cob*, 4—*nad4*, 6—*cox2*, 7—*nad4L*, 8—*nad5*, 9—*atp8*, 10—*atp6*, 12—*cox3*, 16—*nad1*; Archive containing InterProScan analysis results both in *tsv* and *gff3* formats.

## Abbreviations

The following abbreviations are used in this manuscript:

WNS	White-Nose Syndrome
ML	Maximum Likelihood
PCGs	Protein-coding genes
ESS	Effective sample sizes
MCC	Maximum clade credibility
GMST	Global mean surface temperature
ANI	Average Nucleotide Identity
COG	Clusters of Orthologous Genes
Ms	Mississippian epoch
Pn	Pennsylvanian epoch
C	Cisuralian epoch
G	Guadalupian epoch
Lp	Lopingian epoch
ET	Early Triassic epoch
MT	Middle Triassic epoch
LT	Late Triassic epoch
EJ	Early Jurassic epoch
MJ	Middle Jurassic epoch
LJ	Late Jurassic epoch
EC	Early Cretaceous epoch
LC	Late Cretaceous epoch
Pal	Paleocene epoch
Eo	Eocene epoch
Ol	Oligocene epoch
Mio	Miocene epoch
MRCA	Most recent common ancestor
Mya	Million years ago
HPD	Highest posterior density
ATP	Adenosine triphosphate
NADH	Nicotinamide adenine dinucleotide
DNA	Deoxyribonucleic Acid
RNA	Ribonucleic Acid
