# Supplementary figures and images for: Beyond White-Nose Syndrome: Mitochondrial and Functional Genomics of Pseudogymnoascus destructans
Source: J Fungi (Basel). 2025 Jul 24;11(8):550. doi: 10.3390/jof11080550 (PMC12387347; doi:10.3390/jof11080550)

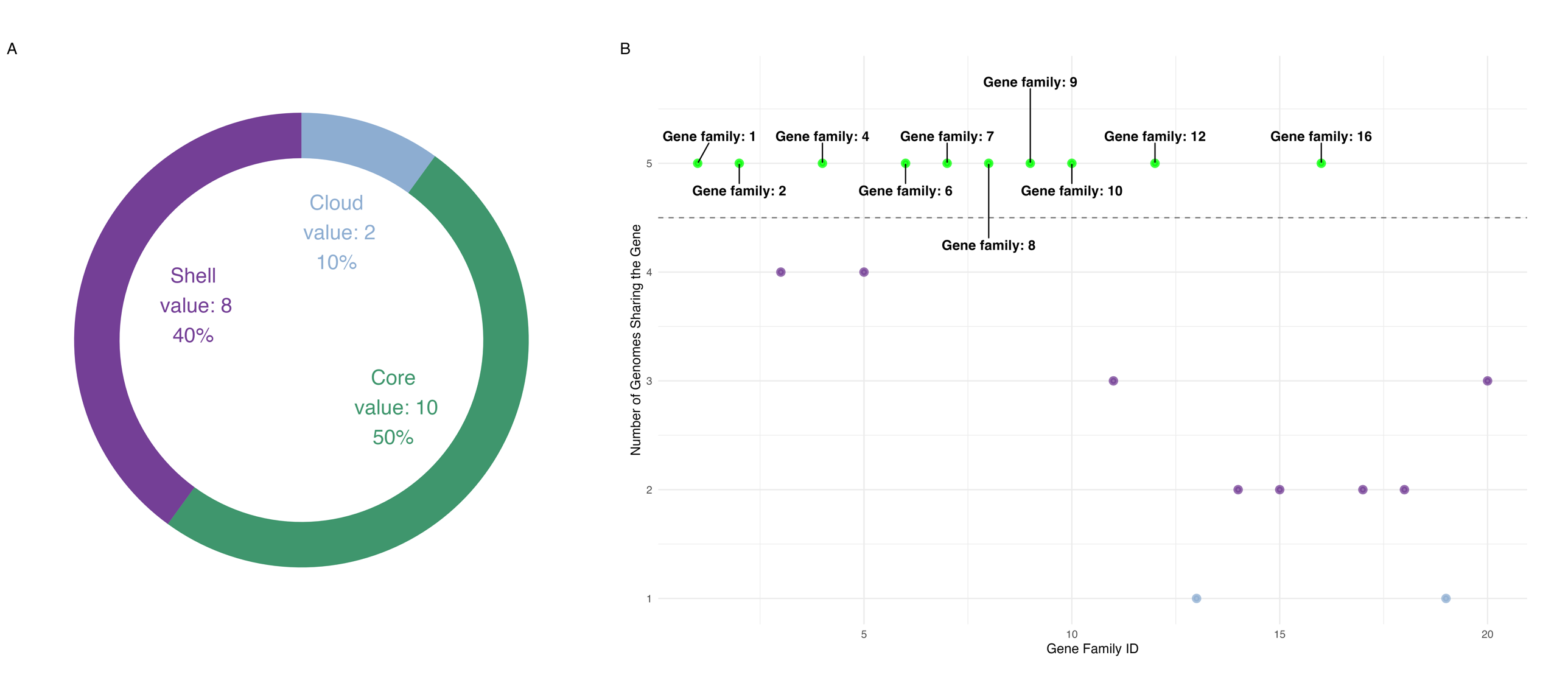

Supplement: Supplementary file 1 [file jof-11-00550-s001.zip › figS2.png]
